# Malignant hilar biliary obstruction with active biliary bleeding: fully covered multi-hole metal stent deployed above papilla

**DOI:** 10.1055/a-2808-7444

**Published:** 2026-03-02

**Authors:** Tsuyoshi Suda, Norihiko Ogawa, Yoshihide Naito, Kenkei Hasatani, Hiroyuki Aoyagi

**Affiliations:** 1 Department of Gastroenterology, Fukui Prefectural Hospital, Fukui, Japan

In patients with malignant hilar biliary obstruction (MHBO), uncovered self-expandable metal stents (SEMSs) are generally not removable, and fully covered SEMSs (FCSEMSs) have the risk of occluding segmental bile ducts 1 . The newly developed multi-hole FCSEMS reduces this occlusion risk, and an increasing number of reports now support its use for MHBO 2 3 .

A 55-year-old man underwent multidisciplinary treatment for rectal cancer, including right hepatic lobectomy and local resection of multiple liver metastases. Subsequently, he developed left hepatic duct stenosis caused by a segment 1 liver metastasis ( Fig. 1 ), for which a plastic biliary stent was placed. However, the stent became occluded, and an endoscopic retrograde cholangiopancreatography (ERCP) attempt at the referral hospital was unsuccessful; therefore, the patient was transferred to our institution.

**Fig. 1:**
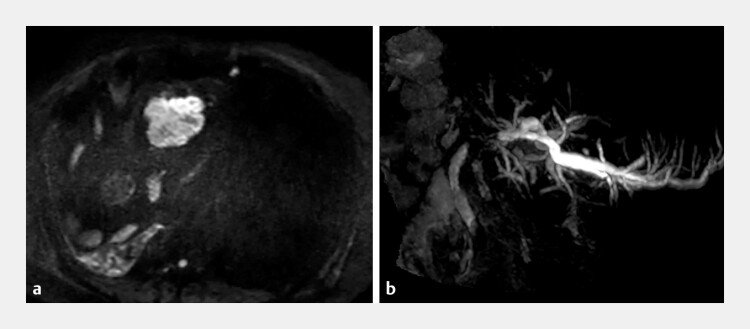
MRCP reveals a metastatic liver tumor in segment 1 **a** and left hepatic duct stenosis **b** . MRCP, magnetic resonance cholangiopancreatography.

At our hospital, a repeat ERCP revealed active bleeding from the tumor ( Fig. 2 ). Because the patient had renal failure, contrast-enhanced computed tomography and magnetic resonance imaging could not be performed, making the preprocedural identification of bleeding difficult. Endoscopic retrograde cholangiography (ERC) showed malignant stenosis of the left hepatic duct owing to tumor involvement ( Fig. 3 ). To decompress the MHBO and control hemobilia, we planned the placement of an FCSEMS. To avoid occluding the segmental ducts, a 10-mm × 8-cm multi-hole FCSEMS (HANARO Biliary Multi-Hole NEO; M.I. Tech Co., Ltd, Pyeongtaek, South Korea) was deployed above the papilla. After the placement of the multi-hole FCSEMS, ERC revealed the segmental ducts without occlusion ( Fig. 4 and Fig. 5 and Video 1 ).

**Fig. 2:**
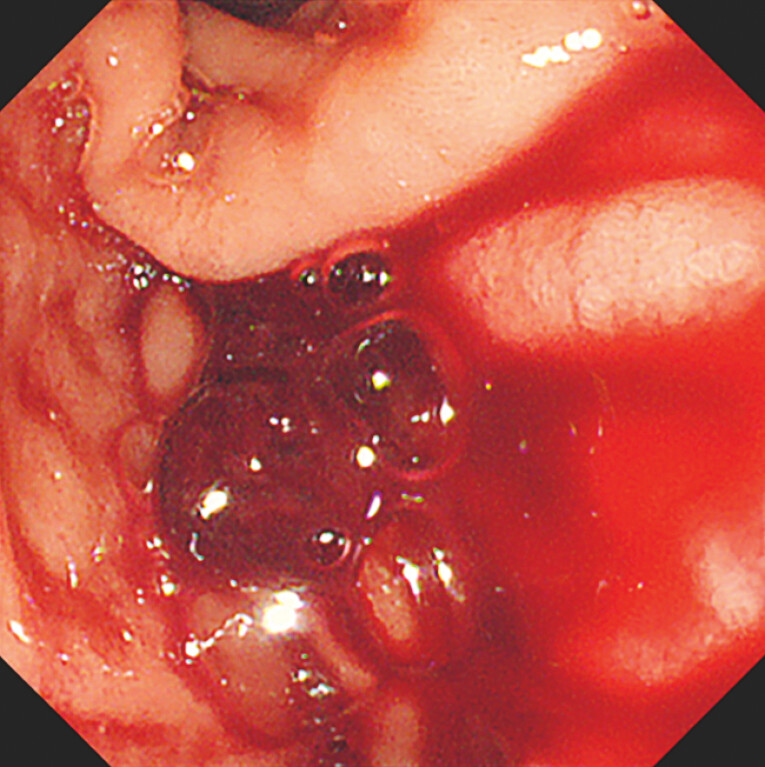
Hemobilia with active bleeding.

**Fig. 3:**
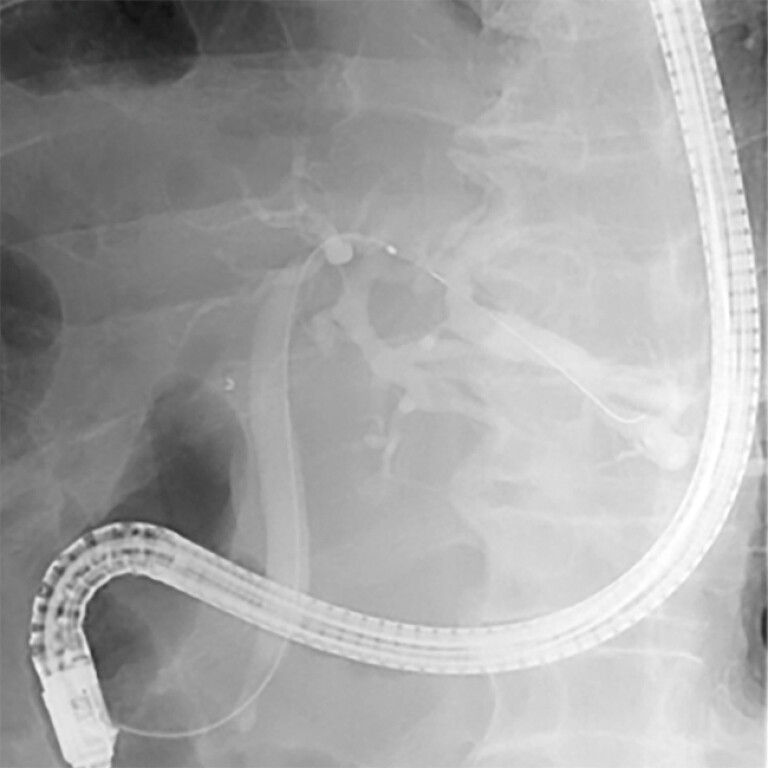
Left hepatic duct stenosis in ERC. ERC, endoscopic retrograde cholangiography.

**Fig. 4:**
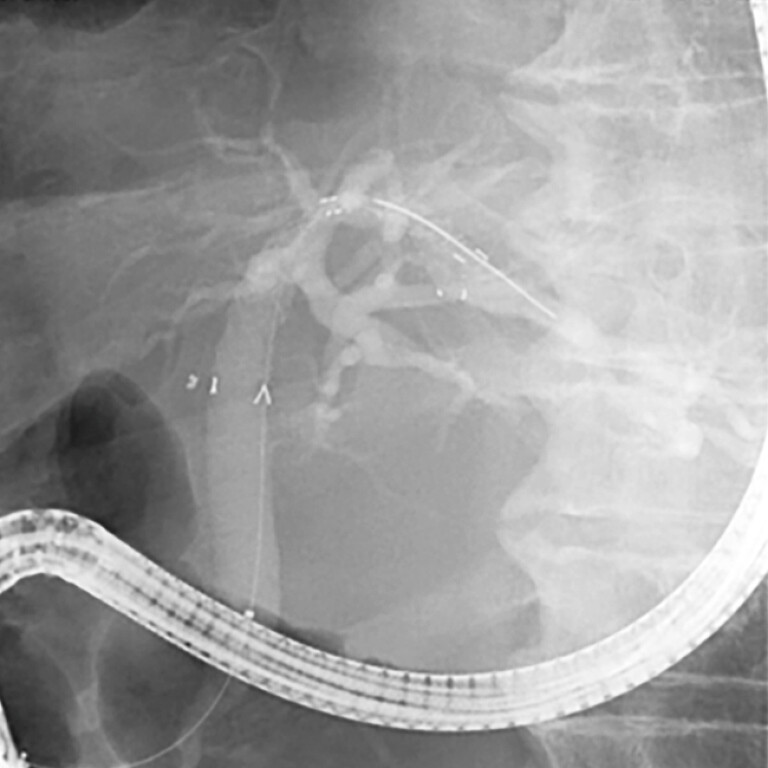
Multi-hole FCSEMS deployed. FCSEMS, fully covered self-expandable metal stents.

**Fig. 5:**
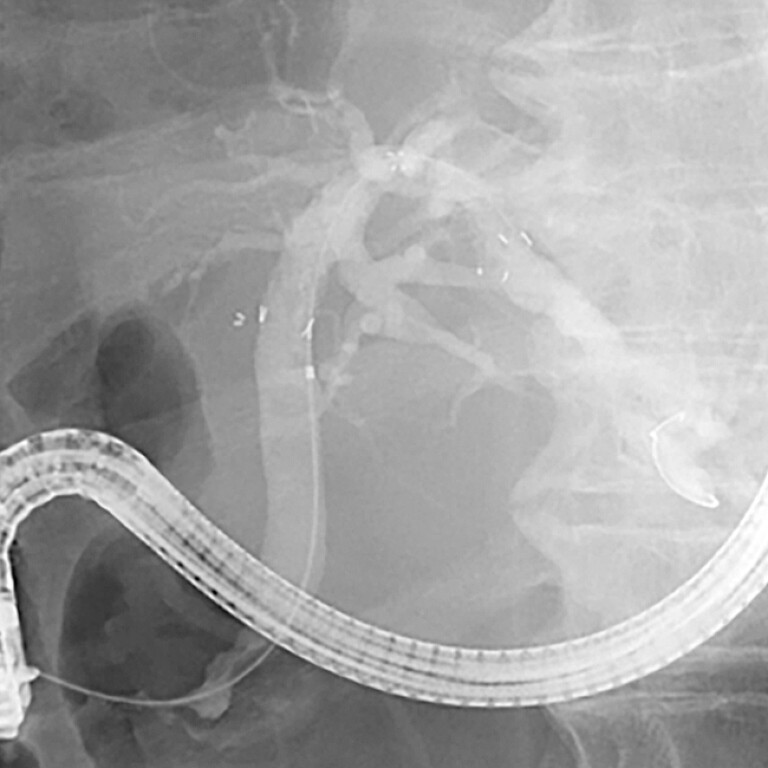
Branches of the bile duct are visualized via multi-hole FCSEMS in ERC. ERC, endoscopic retrograde cholangiography; FCSEMS, fully covered self-expandable metal stents.

Multi-hole FCSEMS reduces the risk of occluding segmental bile ducts in MHBO. FCSEMS, fully covered self-expandable metal stents; MHBO, malignant hilar biliary obstruction.Video 1

Jaundice gradually improved after stent placement. Although hemobilia persisted briefly and necessitated a transfusion, the bleeding resolved upon the complete stent expansion. Approximately 2 weeks after the procedure, the patient was transferred back to the referring hospital.

Successful hemostasis has been reported in a patient with MHBO-related hemobilia through the side-by-side placement of a multi-hole FCSEMS across the papilla 4 . Although there are some concerns regarding the multi-hole design of the FCSEMS, previous studies have reported successful removal 5 .

The multi-hole FCSEMS may therefore serve not only as a novel treatment option for MHBO but also as an effective modality for managing biliary bleeding.

Endoscopy_UCTN_Code_CCL_1AZ_2AC Endoscopy_UCTN_Code_TTT_1AR_2AZ

## References

[1] Dumonceau JM Tringali A Papanikolaou IS Endoscopic biliary stenting: indications, choice of stents, and results: European Society of Gastrointestinal Endoscopy (ESGE) Clinical Guideline – Updated October 2017 Endoscopy 2018 50 910 930 30086596 10.1055/a-0659-9864

[2] Takahashi S Fujisawa T Takasaki Y Side-by-side placement of a novel slim 6-mm multi-hole covered self-expandable metallic stent for malignant hilar biliary obstruction Endoscopy 2025 57 E312 E313 10.1055/a-2569-7582 40233932 PMC12020676

[3] Toyonaga H Oka A Takayama T Triple stent-in-stent placement of novel 6-mm multi-hole covered self-expandable metal stents for malignant hilar biliary obstruction Endoscopy 2025 57 E808 E810 10.1055/a-2641-2204 40719127 PMC12302166

[4] Oishi R Miwa H Maeda S Unique side-by-side technique for malignant hilar biliary obstruction using novel multi-hole self-expandable metallic stents Dig Endosc 2025 10.1111/den.70014 40865927

[5] Ogura T Uba Y Kanadani T Reintervention for recurrent biliary obstruction after stent-in-stent deployment of multi-hole self-expandable metal stents Endoscopy 2025 57 E181 E182 10.1055/a-2534-3143 39978392 PMC11842147

